# Role of immune cells in mediating the effect of gut microbiota on Hashimoto’s thyroiditis: a 2-sample Mendelian randomization study

**DOI:** 10.3389/fmicb.2024.1463394

**Published:** 2024-10-14

**Authors:** Xiao-Qing Pei, Wen-Hao Wang, Yue-Hua Gao, Tong-Xin Zhang, Jing-Yu Liu, Zhen-Dan Zhao, Hua-Wei Zhang

**Affiliations:** Department of Ultrasound, Shandong Provincial Hospital Affiliated to Shandong First Medical University, Jinan, China

**Keywords:** gut microbiota, Hashimoto’s thyroiditis, immune cells, Mendelian randomization, odds ratios

## Abstract

**Purpose:**

Hashimoto’s thyroiditis (HT) is one of the most commonly encountered types of autoimmune thyroid disorders (AITDs), influenced by environmental factors, genetics, and the immune system. Previous research has shown a correlation between gut microbiota and HT, as well as the involvement of immune cells in its onset and progression. We aimed to investigate whether immune cells act as intermediaries in the causal relationship between gut microbiota and HT.

**Methods:**

In this study, we conducted bidirectional two-sample Mendelian randomization (MR) analyses to explore the relationship between gut microbiota and HT using data from genome-wide association studies (GWAS) and the MiBioGen study. Subsequently, MR analyses were performed to investigate the interactions between 731 immune cells and gut microbiota. Additionally, an MR analysis was performed to examine the association between HT and these 731 immune cells, using a GWAS dataset that included 3,757 European subjects. This approach provided insights into the impact of 22 million genetic variants on 731 immune cell signatures.

**Results:**

There was a causal relationship between the increase in the number of 15 gut microbiota and HT. We observed that the genus *Akkermansia*, family Alcaligenaceae, family Desulfovibrionaceae, family Verrucomicrobiaceae, class Verrucomicrobiae, order Verrucomicrobiales, phylum Verrucomicrobia, class Alphaproteobacteria, order Desulfovibrionales, genus *Ruminococcus torques* group, genus *Butyrivibrio*, and genus *Coprococcus3* were negatively correlated with HT. In addition, the genus *Intestinimonas*, genus *Turicibacter*, and genus *Anaerostipes* were positively correlated with HT. We identified EM CD4 + T cells as a mediator between the gut microbiota and HT.

**Conclusion:**

In conclusion, we presented causal associations between the EM CD4 + T cell-mediated gut microbiota and HT, as inferred from the MR findings derived from extensive aggregated GWAS data. Our research offers guidance and direction for treating and preventing HT.

## 1 Introduction

Hashimoto’s thyroiditis (HT) is one of the most common kinds of autoimmune thyroid disorders (AITDs), with pathogenic factors related to environmental factors, genes, and the immune system. It affects approximately 0.3–1.5 cases per 1,000 people (Ragusa et al., 2019; Ralli et al., 2020). It is characterized by chronic inflammation and the presence of autoantibodies targeting thyroid peroxidase (TPO) and thyroglobulin (TG), resulting in hypothyroidism and frequently leading to the degeneration of the thyroid gland (Ragusa et al., 2019; Mikulska et al., 2022). The increasing incidence of HT significantly impacts quality of life, leads to disturbances in thyroid hormone levels, and causes various symptoms in multiple systems, such as depression (Mikulska et al., 2022; Mohammad et al., 2019; Ajjan and Weetman, 2015).

It is reported that the immune system is intricately involved in the occurrence of HT. The immune factors contributing to the onset of HT are associated with abnormal B cell subsets and the production of thyroid autoantibodies, as well as T cell dysfunction that disrupts immune system homeostasis (Du et al., 2022). Furthermore, gut microbiota is associated with the immunological balance in the host. Recent studies have indicated that the absence of Th17 and regulatory T cells (Treg) in mice can be restored through colonization with segmented filamentous bacteria (SBF) and *Clostridium* species, respectively (Virili et al., 2018).

Excluding the immune factors, gut microbiota is also closely associated with the occurrence and progression of HT. The pathogenesis of HT is significantly affected by changes in gut microbiota composition (Virili et al., 2018). On the one hand, dysbiosis, the disruption of the equilibrium and mutualistic relationship between gut microbiota and the host, can increase intestinal permeability, which is associated with HT (Virili et al., 2018). On the other hand, gut microbiota can impact the absorption of minerals that are important for thyroid function (Knezevic et al., 2020).

However, due to the susceptibility of previous studies to confounding factors and reverse causality bias, it has remained unclear whether there is a causal relationship between gut microbiota and HT. It is also uncertain whether immune cells play an intermediary role between the two. Therefore, we conducted this study.

Mendelian randomization (MR) uses genetic variants to investigate the causal relationships between exposure and disease onset (Li et al., 2022). In this study, we utilized the MR method to investigate the causal relationship between gut microbiota and HT, while also exploring the mediating role of immune cells to eventually help offer fresh insights into strategies for both preventing and treating Hashimoto’s thyroiditis (Long et al., 2023).

## 2 Methods

### 2.1 Study design

**Figure 1:**
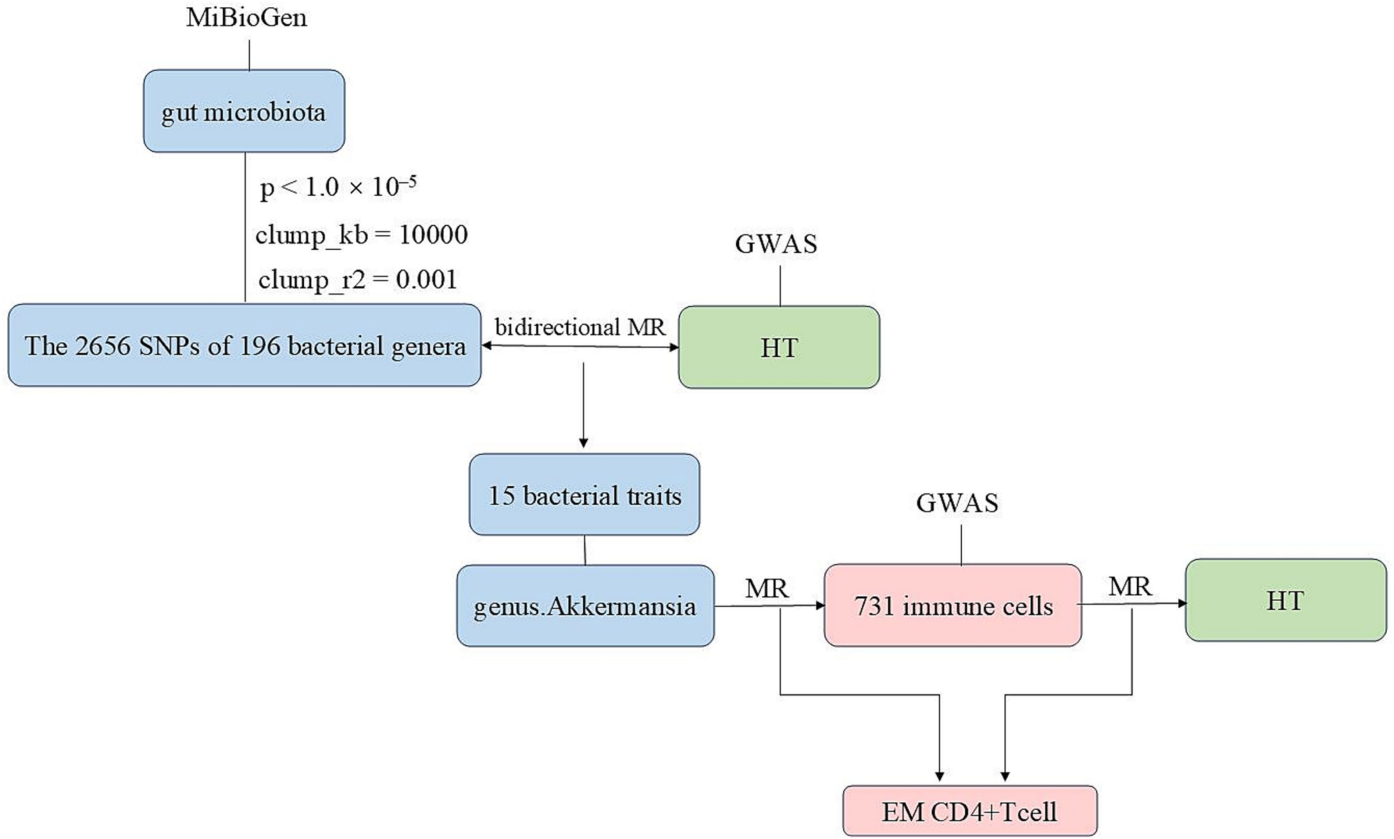
The flowchart of this study illustrating the approach taken to investigate the link between the gut microbiota and Hashimoto’s thyroiditis to determine the causal relationships. Mediation analysis additionally assessed the potential impact of immune cell traits on the gut microbiota—Hashimoto’s thyroiditis association.

We conducted bidirectional two-sample MR analyses using data from genome-wide association studies (GWAS) and the MiBioGen study to explore the relationship between gut microbiota and HT. Subsequently, MR analyses were performed on 731 immune cells and the gut microbiota that exhibited the most significant relationship with HT. This was followed by an MR analysis to examine the association between 731 immune cells and HT. After the aforementioned validation, we ultimately identified immune cells as the mediating factor (Figure 1 and Supplementary Figure S1). Finally, we conducted a mediation analysis, which enabled us to estimate the direct impact of the gut microbiota on HT and calculate the proportion of the immune cells’ influence on HT.

### 2.2 Data sources

Summary genetic data for HT were acquired from a genome-wide association study (GWAS) available at https://gwas.mrcieu.ac.uk/ (ebi-a-GCST90018855), which included 15,654 cases and 3,79,986 controls of European ancestry (Sakaue et al., 2021).

The study sourced summary statistics for gut microbiota from the MiBioGen study, available at https://mibiogen.gcc.rug.nl/menu/main/home/. This extensive research coordinated 18,340 individuals from 24 cohorts, utilizing 16S rRNA gene sequencing profiles (Kurilshikov et al., 2021).

Full GWAS summary statistics for each immune trait are publicly accessible via the GWAS Catalog server at https://www.ebi.ac.uk/gwas/home. The GWAS dataset comprises 3,757 European subjects, providing insights into the impact of 22 million variants on 731 immune cell signatures.

### 2.3 Selection of instrumental variables and clinical diagnostic criteria

We first retained 196 bacterial traits, while removing 15 genus spp. The instrumental variables (IVs) were selected based on the following criteria: (1) Single nucleotide polymorphisms (SNPs) linked to gut bacteria at a significance level of *p* < 1.0 × 10–5 were selected as IVs; (2) we set the linkage disequilibrium (LD) threshold to *R*^2^ < 0.001, with a clumping window size of 10,000 kb in 1000 Genomes EUR data to identify IVs, using “TwoSampleMR” packages; and (3) according to the lowest *p*-value, the SNPs were clumped with the 196 bacterial traits (Verbanck et al., 2018).

We extracted relevant information to calculate F-statistics and *R*^2^ to assess the instrument strength of the selected SNPs. *R*^2^ = (2 × beta^2^)/((2 × beta^2^) + (2 × *N* × SE^2^)), *F* = (*R*^2^ × (*N*−2))/(1−*R*^2^) (Palmer et al., 2012; Kamat et al., 2019).

HT is diagnosed through a combination of clinical features, such as dysphonia, dyspnea, thyroid gland dysfunction, and dysphagia. The diagnostic criteria also include the presence of serum antibodies against thyroid antigens, primarily thyroperoxidase and thyroglobulin. Additionally, thyroid ultrasound findings indicate that the echogenicity of the thyroid parenchyma is significantly reduced, resembling that of the surrounding strap muscles. In fine-needle aspiration cytology, HT presents a polymorphic population of lymphoid cells, with lymphocytes frequently interacting with clusters of thyroid cells (Caturegli et al., 2014).

### 2.4 Statistical analysis

Various methods were employed to investigate the potential causal relationship between HT and gut microbiota, including the MR pleiotropy residual sum and outlier (MR-PRESSO) test, MR-Egger regression, the inverse-variance weighted (IVW) method, and the weighted median method.

The IVW method provided the most accurate estimates of effects, so it was used as the main analysis. The IVW approach merged Wald estimates of individual SNPs to derive the primary causal estimation of the impact of gut microbiota on HT (Bowden et al., 2016). We utilized the MR-Egger regression to assume instrument strength independent of direct effects and to test potential horizontal pleiotropy. This approach makes it possible to evaluate the *p*-value of the intercept; if it is less than 0.05, SNPs may potentially exhibit horizontal pleiotropy (Bowden et al., 2015).

We excluded gut microbiota exhibiting pleiotropy and linkage disequilibrium. We removed significant outliers by performing the MR-PRESSO test to acquire a revised association outcome, thereby reducing bias introduced by pleiotropy (Verbanck et al., 2018). In addition, we utilized the LD trait tool to analyze confounding factors and removed SNPs that might directly influence HT. The associations between the risk of HT and the gut microbiota were expressed as odds ratios (ORs). A *p*-value<0.05 was considered indicative evidence of a potential causal link.

## 3 Results

### 3.1 Exploration of the causal effect of the gut microbiota on HT onset

**Figure 2:**
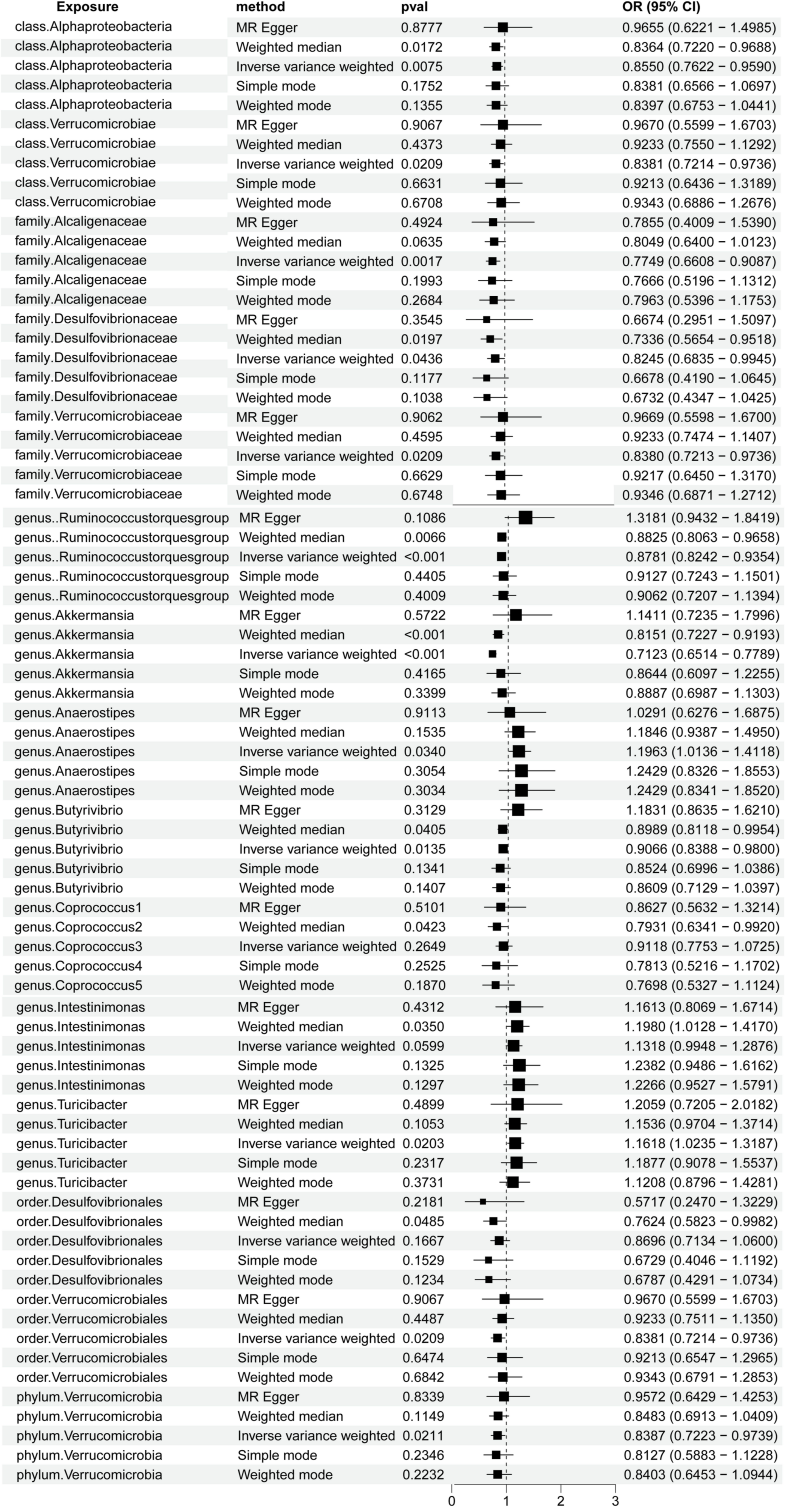
Forest plots demonstrating the causal associations between the gut microbiota and Hashimoto’s thyroiditis using different methods.

A total of 2,656 SNPs of the 196 bacterial genera were selected as IVs based on the selection criteria. Details are presented in Supplementary Table S1. The *R*^2^ values ranged from 0.0009213 to 0.0099786, exhibiting the consistency and dependability of the instrumental variables. The *F* values of all SNPs from the gut microbiota and immune cells exceeded 10. Using multiple methods, we noted direct evidence linking 15 bacterial traits to the risk of HT, as shown in Figure 2. We conducted a screening of the 195 gut microbiota using the criteria of *p* < 1.0 × 10^−8^, kb = 10,000, and *R*^2^ = 0.001. A total of seven gut microbiota were found, each containing only a single SNP, while the remaining gut microbiota did not meet the SNP criteria, rendering the Mendelian randomization analysis infeasible for them. Detailed information on the identified SNPs is provided in Supplementary Table S2.

**Figure 3:**
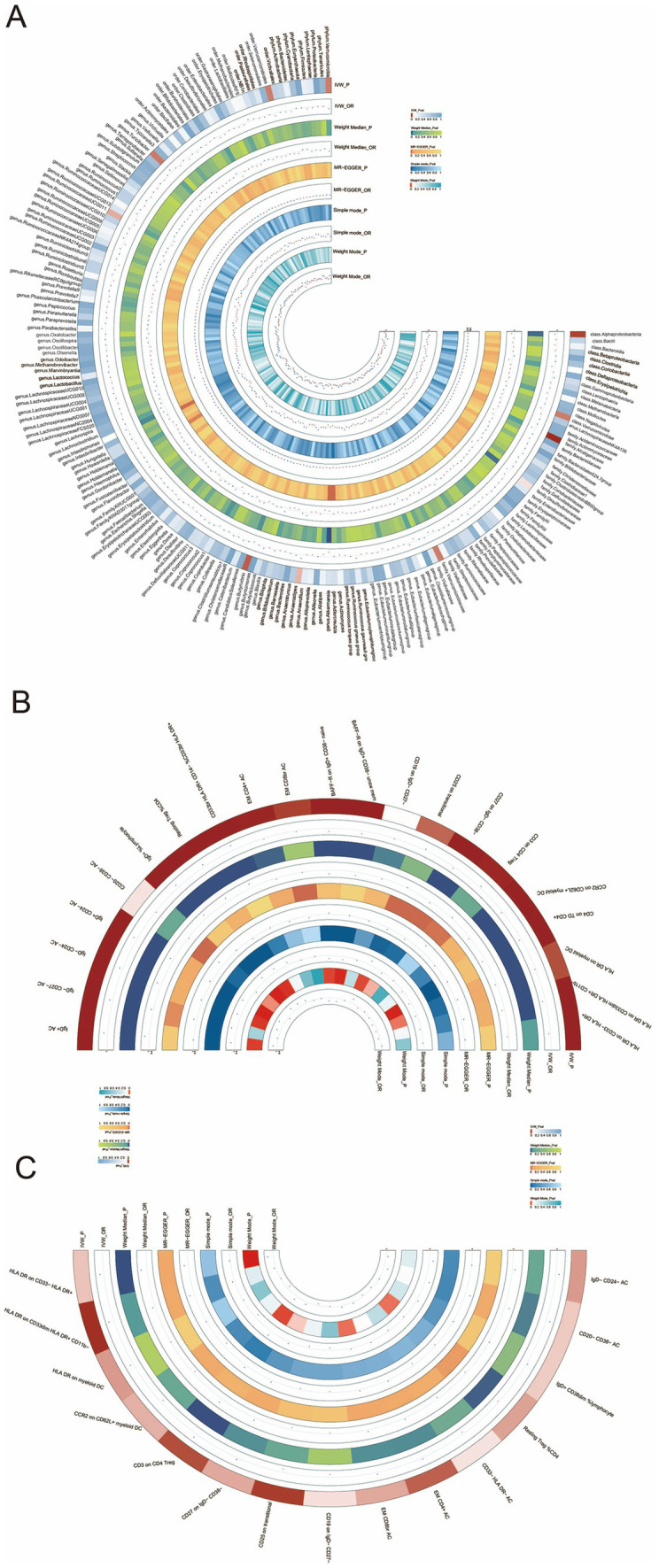
**(A)** Circular heatmap illustrating the relationship between the gut microbiota and Hashimoto’s thyroiditis, **(B)** circular heatmap depicting the relationship between the gut microbiota and immune cells, and **(C)** circular heatmap showing the relationship between Hashimoto’s thyroiditis and the immune cells.

Briefly, the results from multiple methods regarding the connections between all 196 bacterial traits and the risk of HT are shown in Supplementary Table S3 and Figure 3A.

Notably, the genus *Akkermansia* and the genus *Ruminococcus torques* group showed odds ratios (OR) of 0.7123 (*p =* 9.9304E-14) and 0.8781 (*p =* 0.0001) in the IVW method, respectively. Among the gut microbiota in the IVW method, the family Alcaligenaceae showed an OR of 0.7749(*p* = 0.0017), class Alphaproteobacteria showed an OR of 0.8550 (*p* = 0.0075), genus *Butyrivibrio* showed an OR of 0.9066 (*p* = 0.0135), genus *Turicibacter* showed an OR of 1.1618 (*p* = 0.0203), family Verrucomicrobiaceae showed an OR of 0.8380 (*p* = 0.0209), order Verrucomicrobiales showed an OR of 0.8381 (*p* = 0.0209), class Verrucomicrobiae showed an OR of 0.8381 (*p* = 0.0209), phylum Verrucomicrobia showed an OR of 0.8387 (*p* = 0.0211), genus *Anaerostipes* showed an OR of 1.1963 (*p* = 0.0340), and family Desulfovibrionaceae showed an OR of 0.8245 (*p* = 0.0436).

The MR-PRESSO analysis did not identify any statistically significant outliers (Supplementary Table S4). However, potential outliers were observed upon visual inspection of scatter plots (Supplementary Figure S1) and leave-one-out plots (Supplementary Figure S2).

Furthermore, the OR for the gut microbiota in the weighted median method showed that order Desulfovibrionales exhibited an OR of 0.7624 (*p* = 0.0485), genus *Coprococcus1* exhibited an OR of 0.7931 (*p* = 0.0423), and genus *Intestinimonas* exhibited an OR of 1.1980 (*p* = 0.0350). We genetically predicted that specific constituents of the gut microbiota were associated with the risk of HT. Using the MR-PRESSO, we detected no outliers, further solidifying the association between HT and the gut microbiota. The MR-Egger regression showed all intercepts with *p* > 0.05, implying no significant pleiotropy.

In the reverse MR analysis, HT did not significantly affect gut microbiota composition, as shown in Supplementary Table S5.

### 3.2 Exploration of the mediator of the gut microbiota and HT

To explore the mediating role of immune cells in both gut microbiota and HT, we selected the *genus Akkermansia* for analysis as it exhibits the most significant relationship between gut microbiota and HT. MR analyses were conducted separately for the gut microbiota and immune cells, followed by an MR analysis for the immune cells and HT. The key mediating factor, EM CD4 + T cell, was identified. The relationships between the immune cells, the gut microbiota, and HT are illustrated in Figures 3B,C, as well as in Supplementary Tables S6, S7.

We genetically predicted that the *genus Akkermansia* was positively associated with the risk of EM CD4 + T cells (OR: 0.8189; 95% confidence interval (95%CI) = 0.7273–0.9221; and *p* = 0.0010) using the IVW method. The association between the EM CD4 + T cells and HT remained steady (OR: 1.0294; 95%CI = 1.0058–1.0535; and *p* = 0.0143) in the IVW method. The robustness of the results was assessed using the MR-PRESSO test. Supplementary analyses are displayed in Supplementary Table S8.

When the EM CD4 + T cells acted as the mediator between the gut microbiota and HT, we found that the gut microbiota were associated with the increased EM CD4 + T cells, which in turn correlated positively with an elevated risk of HT. This was evidenced by a direct effect of −0.3335 and a total effect of −0.3393. Further details, including the proportion of the mediating effect, standard error, and *Z* statistic, are presented in Supplementary Table S9.

## 4 Discussion

Our MR analyses strongly support a causal link between gut microbiota and HT. Notably, genera such as EM CD4 + T cells become a key mediator in this relationship.

The *genus Akkermansia* is one of the most common gut microbiota genera, which plays a pivotal role in the development of HT. The current study found that this genus comprises a single member, designated as *Akkermansia muciniphila*, which is notably abundant in patients with HT. Furthermore, research has shown that the relative abundance of the *genus Akkermansia* is higher in patients with HT compared to the control group (Liu et al., 2022).

It is therefore likely that such negative connections exist between the *genus Akkermansia* and HT, implying that the *genus Akkermansia* plays a protective role for patients with HT. Research has shown that the *genus Akkermansia* interacts with cells and is involved in immune signal transduction, contributing to the synthesis of various inflammatory markers, which leads to reduced expression of IL-6 and TNF-*α*. Studies have indicated that serum concentrations of IL-6 are higher in patients with HT compared to normal individuals, and TNF-α exhibits cytotoxicity and inhibits cell growth in thyroid cells (Siemińska et al., 2010; Molaaghaee-Rouzbahani et al., 2023; Baki et al., 2012; Cani et al., 2022). Our finding aligns with recent studies indicating that the *genus Akkermansia* may influence the development of HT through inflammatory factors such as IL-6 and TNF. Based on these data, we can infer that the quantities of the *genus Akkermansia* and EM CD4 + T cells exhibit an inverse relationship. However, this result has not previously been reported. Our findings are in agreement with experiments demonstrating that introducing the *genus Akkermansia* in the form of extracellular vesicles can increase the number of regulatory T cells (Treg), potentially leading to an imbalance in Th17/Treg cells, which can result in damage to CD34+ cells (Kim et al., 2024; Ni et al., 2022). Furthermore, previous research has indicated a correlation between higher doses of transplanted CD34+ cells and increased counts of EM CD4+ T cells (Politikos et al., 2020). Therefore, a possible explanation for this might be that CD34+ cells and Treg cells may represent potential mechanisms underlying the reduction in EM CD4 + T cell numbers caused by the *genus Akkermansia*.

This observation supports the hypothesis that the positive correlation between HT and EM CD4 + T suggests that EM CD4 + T cells are a risk factor for the occurrence and progression of HT. IFN- g is an inflammatory factor from EM CD4 + T cells, participating in killing infected cells (Mehta et al., 2018; Mueller et al., 2008). IFN- g plays a critical role in regulating the host’s defense system by mediating both innate and adaptive immune responses, its signaling pathway is closely linked to cell-mediated immune reactions and inflammation (Bhat et al., 2018). IFN-g-dependent chemokines are considered biomarkers for thyroid inflammation, associated with more severe characteristics that contribute to hypothyroidism and thyroid damage (Ragusa et al., 2019). From the discussion, we conclude that EMCD4 + T cells play a certain role in the occurrence and development of HT through the inflammatory factor IFN-g.

We describe the results suggesting that there is a positive correlation between the *genus Turicibacter* and HT, as well as between the *genus Anaerostipes* and HT, indicating that both *genus Turicibacter* and *genus Anaerostipes* are risk factors for patients with HT. The current study found that the gut microbiota *genus Turicibacter* and *genus Anaerostipes* are both associated with bile acids and lipid metabolism and that HT is commonly associated with dysregulated lipid metabolism. Animal studies have shown that the *genus Turicibacter* can alter the host’s bile acids and lipid metabolism (Lynch et al., 2023). Another important finding was that the *genus Anaerostipes* has adverse effects on the host through the synthesis of fatty acids (Liu et al., 2024). Therefore, we propose two types of mechanisms for the *genus Turicibacter* and *genus Anaerostipes*, which lead to HT via bile acids and lipid metabolism. The first mechanism suggests that phospholipids can mediate cellular stress and inflammation, potentially leading to HT. The alternative mechanism suggests that fatty acid degradation and fatty acid oxidation primarily involve the development and differentiation of Treg cells, further affecting the imbalance between T helper 17 (Th17) cells and Treg cells, thus leading to the occurrence of HT (Liu et al., 2014; Miao et al., 2022). In addition, fatty acid oxidation may contribute to the onset of HT by stimulating the release of inflammatory mediators and altering the function and differentiation of T cells (Jiang et al., 2022). In summary, the gut microbiota *genus Turicibacter* and *genus Anaerostipe* potentially influence the occurrence of HT through bile acids and lipid metabolism, as well as immune cells.

We explored HT from the perspectives of gut microbiota and immune cells, providing the first comprehensive validation and exposition of the interrelations among them. The MR-PRESSO approach was used to identify and correct for outlier pleiotropic instruments by detecting and removing anomalous instrumental variables, thereby reducing bias introduced by pleiotropy. Although our study has revealed important discoveries, there are also limitations. First, the data for HT originates from Europe, thus it cannot be directly inferred whether similar causal relationships exist in other regions. Second, animal and clinical studies were not conducted to directly establish the causal connection between immune cell-mediated HT and gut microbiota. Third, when assessing the gut microbiota, measurement bias might have arisen from the technical factors, sample handling, or differences in laboratory methods. This could have affected the measurement of the exposure variables and led to bias in the MR analysis results. Finally, the 16S rRNA sequencing used in the MiBioGen consortium’s GWAS data on gut microbiota can only detect genetic information at the genus to phylum level, with no data available at the species level. Additional genetic techniques, such as whole genome sequencing or nanopore technology, could enhance our research.

## 5 Conclusion

In conclusion, we presented causal associations between the immune cell-mediated gut microbiota and HT, as inferred from the MR findings derived. Our research offers guidance and direction for the clinical treatment and prevention of HT. Through our findings, we aim to contribute to the advancement of knowledge in the field of HT.

## Data Availability

The original contributions presented in the study are included in the article/Supplementary material, further inquiries can be directed to the corresponding authors. All packages for data analysis used in this study were open source in R software (version 4.3.1; R Development Core Team).
