# Supplementary material for: Role of immune cells in mediating the effect of gut microbiota on Hashimoto’s thyroiditis: a 2-sample Mendelian randomization study
Source: Front Microbiol. 2024 Oct 14;15:1463394. doi: 10.3389/fmicb.2024.1463394 (PMC11513624; doi:10.3389/fmicb.2024.1463394)
Supplement: Supplementary file 5 [file Table_5.DOCX]

Supplementary Material

# Supplementary Figures and Tables

## Supplementary Figures


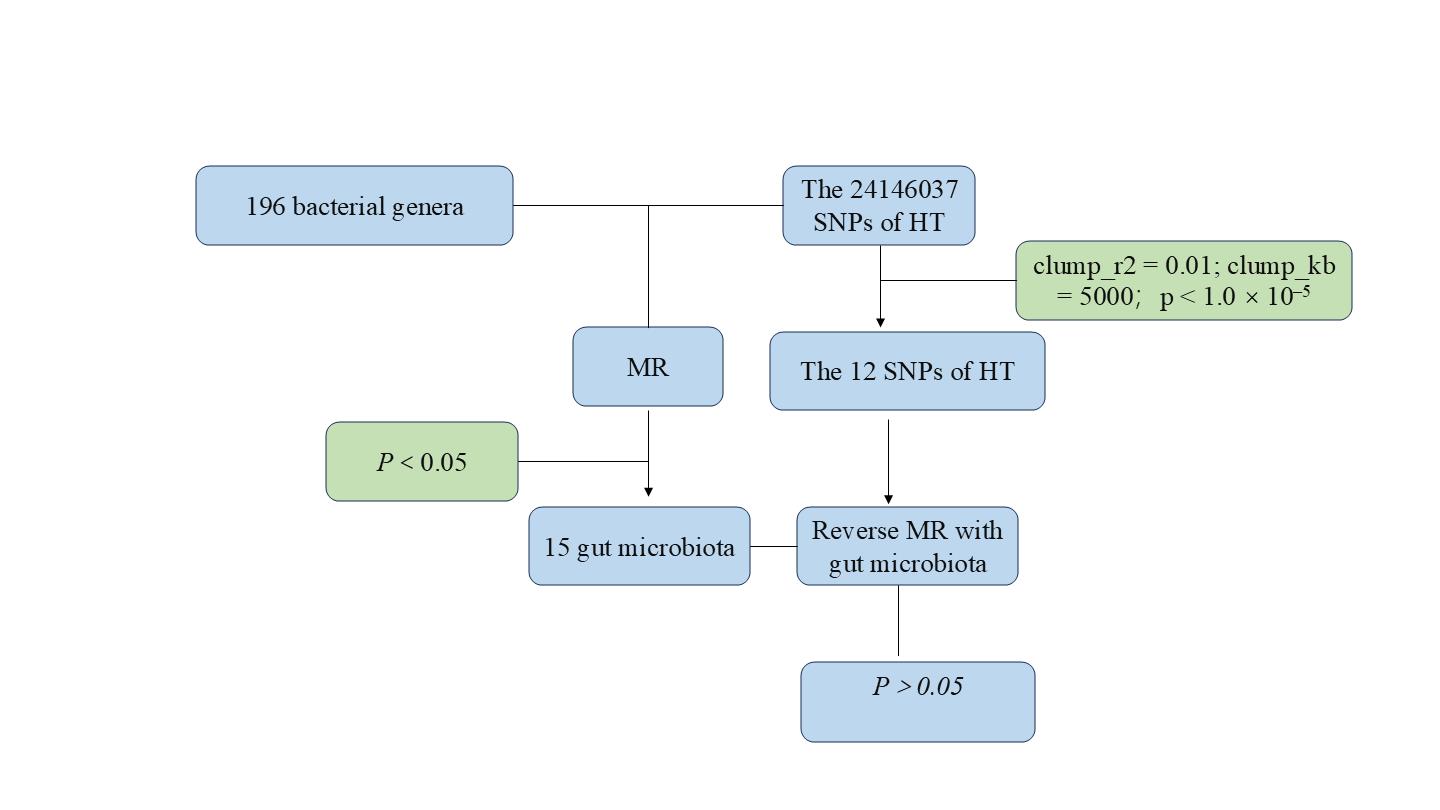


**Supplementary Figure 1**: A flowchart of the disease selection process and its results.


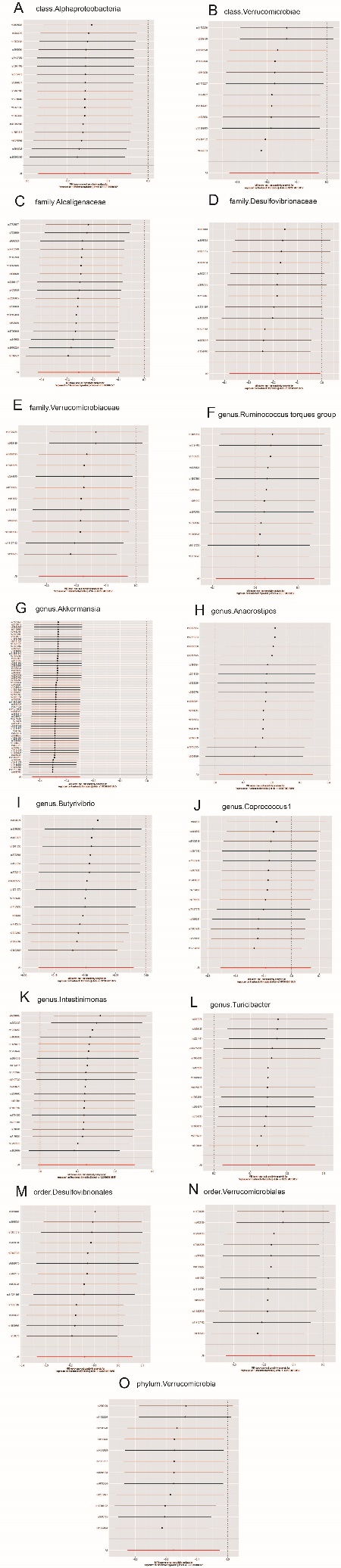


**Supplementary Figure 2**: Scatter plots for the causal association between gut microbiota and Hashimoto's thyroiditis.


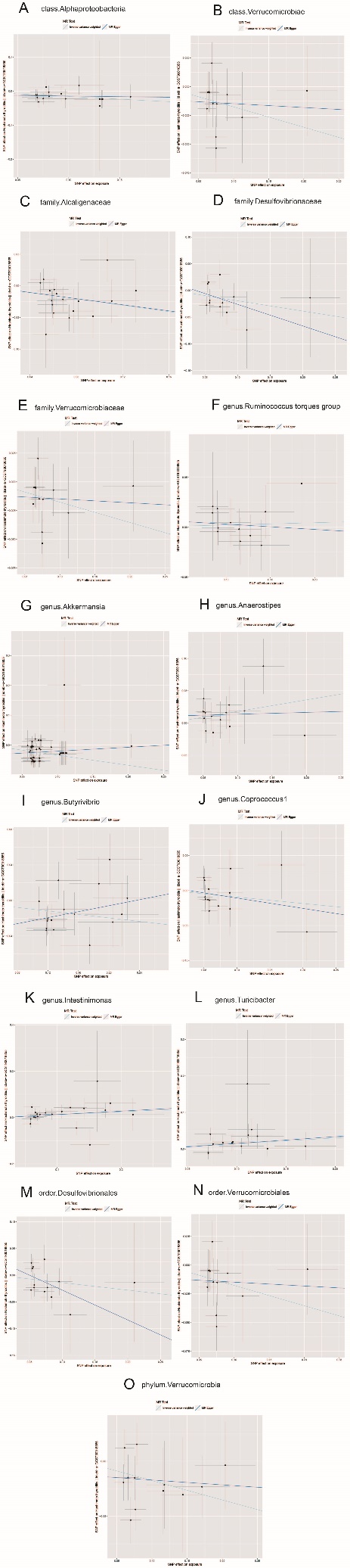


**Supplementary Figure 3**: Leave-one-out plots for the causal association between gut microbiota and Hashimoto's thyroiditis.

##
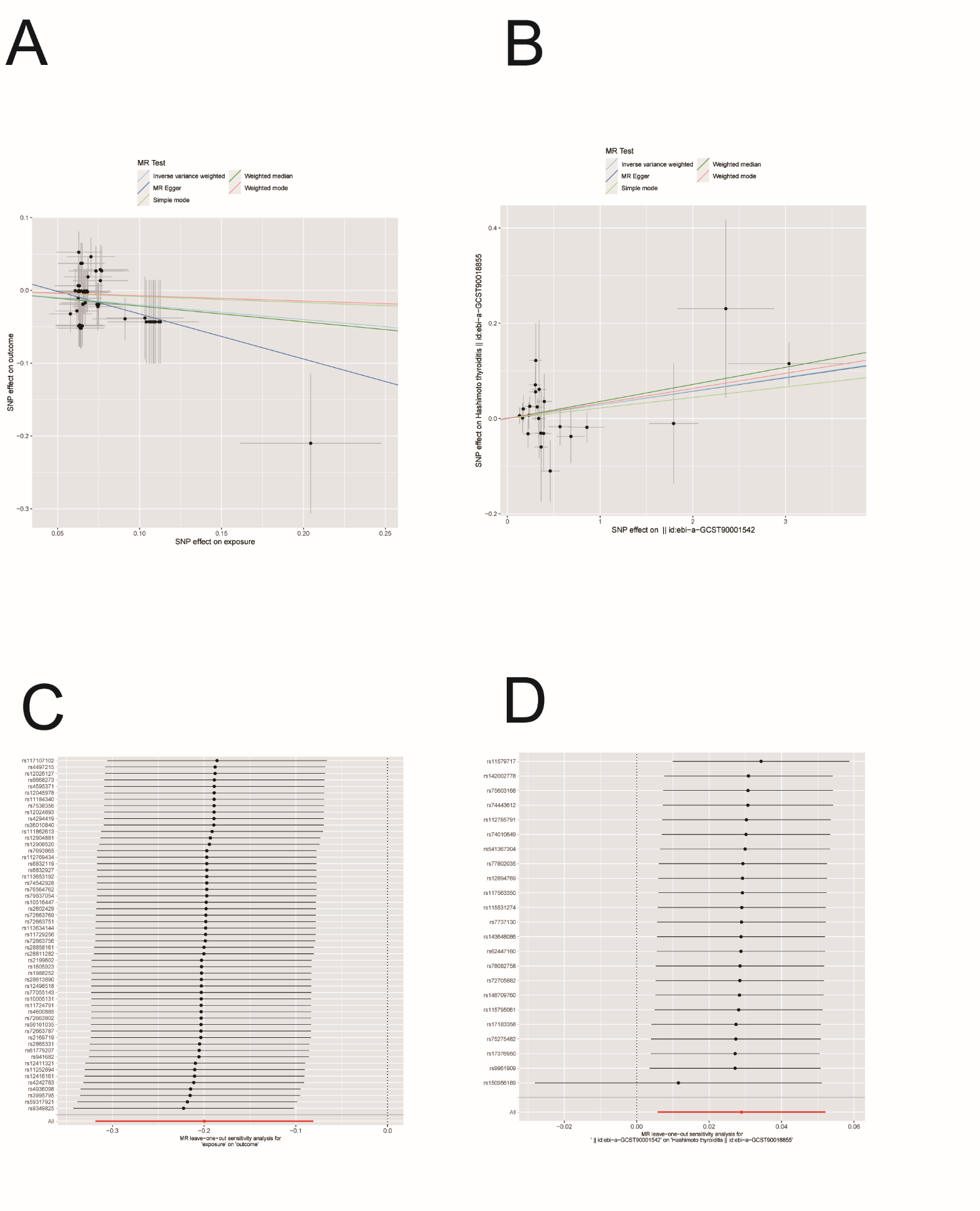


**Supplementary Figure 4.** A: Scatter plots for the causal association between Genus.Akkermansia and EM CD4+T cells. B: Scatter plots illustrating the causal relationship between EM CD4+ T cells and Hashimoto's thyroiditis. C: Leave-one-out plots for the causal association between EM CD4+T cells and Genus.Akkermansia. D: Leave-one-out plots depicting the causal relationship between EM CD4+ T cells and Hashimoto's thyroiditis.
