# Supplementary material for: Role of immune cells in mediating the effect of gut microbiota on Hashimoto’s thyroiditis: a 2-sample Mendelian randomization study
Source: Front Microbiol. 2024 Oct 14;15:1463394. doi: 10.3389/fmicb.2024.1463394 (PMC11513624; doi:10.3389/fmicb.2024.1463394)
Supplement: Supplementary file 11 [file Table_11.DOCX]

<https://github.com/pxqp01425/Raw-data-and-code/blob/247748b643b845f8a0a66e1c50f8c3359219acfb/Raw%20data%20and%20code.zip>
